# miR-152/TNS1 axis inhibits non-small cell lung cancer progression through Akt/mTOR/RhoA pathway

**DOI:** 10.1042/BSR20201539

**Published:** 2021-01-04

**Authors:** Jinjin Duan, Li Wang, Liqun Shang, Shumei Yang, Hua Wu, Yongcheng Huang, Yi Miao

**Affiliations:** 1 Department of Respiratory and Critical Care Medicine, Shaanxi Provincial People’s Hospital, Xi’an, Shaanxi 710068, P.R. China; 2 Department of Pathology, Xi’an Central Hospital, Xi’an, Shaanxi 7100033, P.R. China

**Keywords:** Akt/mTOR/RhoA pathway, growth, metastasis, miR-152, NSCLC, TNS1

## Abstract

*Aim*: The purpose of the present study was to explore the function and mechanism of tensin 1 (TNS1) in non-small cell lung cancer (NSCLC) progression.

*Methods*: The expression of TNS1 in NSCLC cells and tissues was assessed by RT-PCR and Western blot. Besides, Kaplan–Meier survival analysis was recruited to explore the association between TNS1 and NSCLC. Cell growth was analyzed by MTT and flow cytometry assay, while cell metastasis was determined by wound healing and transwell assays. The targeting relationship between TNS1 and miR-152 was assessed by luciferase activity assays. And Western blot was employed to determine the expression of related proteins of Akt/mTOR/RhoA pathway.

*Results*: TNS1 level was boosted in NSCLC cells and tissues, related to the prognosis of NSCLC patients. Furthermore, it was proved that TNS1 promoted the growth and metastasis of NSCLC cells via Akt/mTOR/RhoA pathway. And miR-152 targeted TNS1 to affect the progression of NSCLC.

*Conclusion*: miR-152/TNS1 axis inhibits the progression of NSCLC by Akt/mTOR/RhoA pathway.

## Introduction

Lung cancer is one of the dominant causes of cancer-related deaths worldwide [1], among which non-small cell lung cancer (NSCLC) is the dominant histological type that accounts for ∼80% [2]. Though the prognosis of lung cancer patients at all stages has improved in recent decades, the clinical treatment effect is not satisfactory [3]. The development of molecular-targeted drugs is the hope to improve the overall survival and quality of life of cancer patients [4], while the exact molecular mechanism of lung cancer is still unclear. Thus, finding novel and promising target genes is urgent for exploring an effective treatment for NSCLC.

Tensin is a group of multidomain scaffold proteins, involved in various signaling pathways [5]. Tensin 1 (TNS1), as a member of tensin family, has been reported to function as a scaffold for adhesion-related signaling through binding to actin cytoskeleton and β1-integrin [6]. Importantly, TNS1 participated in the development of many tumors [7–9]. For example, transgelin/TNS1 axis advanced proliferation and invasiveness of colorectal cancer cells [8]. Down-regulated TNS1 suppresses the proliferation of acute myeloid leukemia cells and increases the apoptosis [10]. Furthermore, the expression of TNS1 is boosted during pulmonary fibrosis and is critical for myofibroblast differentiation and extracellular matrix formation [11]. TNS1 has been proved to be up-regulated in lung cancer cells following (-)-epigallocatechin-3-gallate treatment which was a potential anticancer agent [12]. However, the role of TNS1 in NSCLC tumorigenesis is still unclear.

MicroRNAs (miRNAs) are small non-coding RNAs that regulate various cellular processes by binding to the 3′-untranslated region (3′-UTR) of target mRNAs, leading to mRNAs degradation or inhibiting mRNAs translation [13]. More and more reports showed that miRNAs played key roles in regulating initiation and progression of cancers [14,15]. According to the cancer variety, miRNAs could function as oncogenes or tumor suppressors [16–18]. miRNAs dysfunction happened in multiple cancers, including NSCLC, and was related to the tumorigenesis [19,20]. miR-152 plays an anti-tumor role in many cancers [20,21], and is down-regulated in NSCLC [22]. Overexpressing miR-152 could suppress proliferation, colony formation, migration and invasion of NSCLC cells [23].

In the present study, TNS1 was significantly elevated in NSCLC cells and tissues, and was negatively correlated with poor prognosis. And TNS1 promoted the progression of NSCLC by Akt/mTOR/RhoA pathway. In addition, it was found that miR-152 directly targeted TNS1 in NSCLC cells. And miR-152 was negatively correlated with TNS1 in NSCLC tissues, having adverse effects on the TNS1 pro-NSCLC function. In summary, these results indicate that TNS1 may be a therapeutic and prognostic target for NSCLC treatment.

## Materials and methods

### Patient samples and cell lines

Human NSCLCs and adjacent normal tissues were gathered from 36 patients with informed written consent, approved by the Medical Ethics Committee of Shaanxi Provincial People’s Hospital. All tissue specimens were stored in liquid nitrogen for following experiments. The NSCLC cell lines A549, H460, SPCA1, SK-MES-1 and H1299 and the normal lung bronchus epithelial cell line 16HBE were purchased from ATCC (Manassas, VA, U.S.A.) and maintained in Dulbecco’s modified Eagle’s medium with 10% fetal bovine serum in a humidified, 5% CO_2_ atmosphere at 37°C. After reaching 80% confluence, miR-152 mimic/inhibitor was purchased from RiboBio (Guangzhou, China). NSCLC cells were transfected with si-NC, si-TNS1, mimic NC/miR-152 (50 nM), miR-152 inhibitor/inhibitor NC (50 nM) by Lipofectamine 2000 (Invitrogen, Carlsbad, CA, U.S.A.).

### Luciferase activity assays

A total of 50% confluent HEK293 cells were co-transfected with 100 ng wild-type or mut TNS1 3′-UTR and 100 nM miR-152 or control mimics, 20 ng pRL-TK and 100 ng pGL3 control vector though Lipofectamine 2000. Mutation of the TNS1 3*′*-UTR (Mut) was obtained using a mutation kit (Stratagene, La Jolla, CA, U.S.A.). Cells were harvested after 48 h transfection and luciferase activity was determined by dual-luciferase reporter assay system (Promega). Results were normalized to the *Renilla* luciferase.

### RNA extraction and quantitative real-time PCR

Total RNA was collected by TRIzol (Invitrogen) and miR-152 was isolated by All-in-One microRNA extraction kit (GeneCopoeia, Carlsbad, CA, U.S.A.) and detected by All-in-One miRNA qRT-PCR Detection Kit (GeneCopoeia, Carlsbad, CA, U.S.A.). Primers were as follows, TNS1, 5′-TCAAGTGGAAGAACTTGTTTGCTT-3′ (forward) and 5′-CACGACAATATAGTGGAGGCACA-3′ (reverse); TNS1 expression was normalized to glyceraldehyde 3-phosphate dehydrogenase (GAPDH) and miR-152 was normalized to U6. Quantitative real-time PCR (qRT-PCR) was implemented using SYBR Green reagents (TAKARA, Tokyo, Japan). Expression levels were quantified with 2^−ΔΔ*C*_t_^ method.

### Cell proliferation assay

Transfected cells were seed into 96-well plates (4000 cells/well) and incubated for different times. A 10-μl MTT was added to each well and cultured for 4 h. Then the supernatant was abandoned and 200 μl DMSO was added into each well. Optical density (OD) was analyzed at the wavelength of 490 nm.

### Apoptosis detection assay

Annexin V-fluorescein isothiocyanate (FITC) Apoptosis Detection Kit (NanJing KeyGen Biotech Co., Ltd, Nanjing, China) was used to analyze the level of cell apoptosis. In brief, 5 × 10^5^ cells were washed with PBS and resuspended in 400 μl binding buffer. Then, propidium iodide and FITC-conjugated Annexin V were used to stain cells for 1 h in dark. At last, the results were analyzed with a flow cytometry through Cell Quest 3.3 software (FACScan, BD, United States).

### Wound healing assay

Cells were seeded into six-well plates. After 24 h, pipette tips were used to create three scratch wounds, scratching the cell monolayer, and then, cells were incubated for another 24 h. At last, pictures of scratch were taken under microscope and the motility of cells were compared by the degree of confluence of the scratch from one side to the other.

### Cell invasion assay

After 48-h transfection, cells were placed in the upper chamber of 24-well transwell inserts (aperture 8 mm) with serum-free medium and pre-coated with Matrigel. At the same time, medium containing 10% FBS was added into the lower chamber as a chemoattractant. After incubation at 37°C for 24 h, non-invading cells at the top side of the inserts were taken out by cotton swabs while invading cells on the lower membrane were fixed and stained with 0.1% Crystal Violet dye. At last, stained cells were detected and counted under an inverted microscope.

### Western blotting

RIPA was used for protein extraction (Beytime, Shanghai, China). Proteins were isolated with 10% SDS/PAGE, transferred on to nitrocellulose membranes and blocked for 1 h by TBS containing 5% skim milk. Primary antibodies were used for incubation of members overnight at 4°C, including anti-TNS1 (1:1000, ab167660, Abcam), anti-E-cadherin (1:2000, SC-71008, Santa Cruz Biotechnology), anti-N-cadherin (1:2000, SC-59987, Santa Cruz Biotechnology), anti-vimentin (1:2000, SC-32322, Santa Cruz Biotechnology), anti-RhoA (1:2000, SC-418, Santa Cruz Biotechnology), anti-Akt (1:2000, SC-56878, Santa Cruz Biotechnology), anti-phospho-Akt (1:2000, SC-377556, Santa Cruz Biotechnology) and GAPDH (1:800, 5174, Cell Signaling Technology). Then, members were maintained with secondary antibodies at room temperature for 1 h. An enhanced chemiluminescence (ECL) system (Pierce Biotechnology, Rockford, U.S.A.) was recruited to visualize the bound antibodies. GAPDH was employed as a loading control.

### RhoA activation assay

Cells were washed with cold PBS and dissolved. Equal protein volume of cell lysates was cultured with 30 μg glutathione S-transferase-Rho binding domain of rhotekin (GST-RBD) beads (Cytoskeleton) for 1 h at 4°C, followed by two washes in the washing buffer. Bound Ras homolog (Rho) proteins were collected and determined with 12% SDS/PAGE for Western blotting analysis.

### Statistical analysis

Data were shown as the mean ± S.E.M. Statistical analyses were implemented with Student’s *t* test or one-way ANOVA by SPSS 12.0 (Chicago, IL, U.S.A.). *P*<0.05 was considered statistically significant.

## Results

### High expression of TNS1 is related to patient prognosis

**Figure 1:**
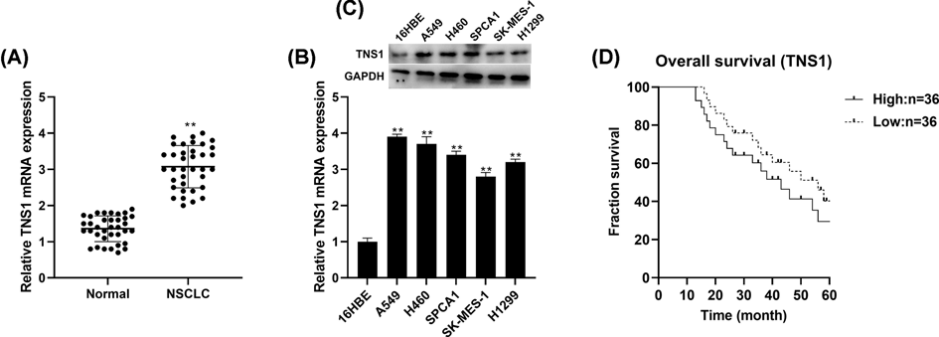
TNS1 expression is increased in NSCLC tissues and cells (**A**) By qRT-PCR, TNS1 levels in NSCLC tissues and adjacent normal lung tissues from 36 patients were determined. TNS1 mRNA (**B,C**) protein expression were detected through RT-PCR in different NSCLC cells. (**D**) Correlation between TNS1 levels and overall survival was assessed by Kaplan–Meier survival analysis. **P<0.01 compared with adjacent normal lung tissues or 16HBE cells.

TNS1 expression in NSCLC tissues was significantly higher, compared with adjacent normal lung tissues (Figure 1A). mRNA and protein level of TNS1 were also up-regulated in NSCLC cells, A549, H460, SPCA1, SK-MES-1 and H1299 compared with normal lung epithelial 16HBE cells (Figure 1B,C). Furthermore, Kaplan–Meier survival analysis showed that high TNS1 expression was significantly correlated with poor prognosis in patients with NSCLC (Figure 1D).

### TNS1 promotes the growth of NSCLC cells

**Figure 2:**
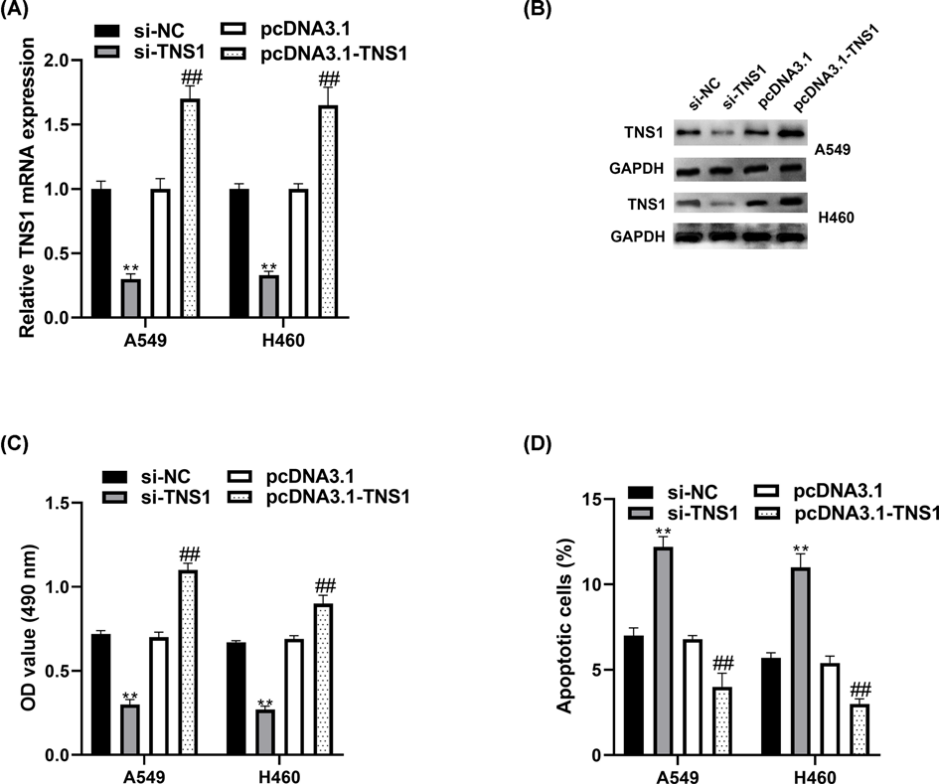
TNS1 regulates NSCLC cells growth A549 and H460 cells were transfected with si-NC or si-TNS1. (**A,B**) TNS1 expression was determined through RT-qPCR and Western blot. (**C,D**) Effect of TNS1 on cell proliferation and apoptosis was assessed by MTT assay and flow cytometry. **P<0.01 compared with si-NC. ^##^*P*<0.01 compared with pcDNA3.1.

To figure out whether TNS1 regulates NSCLC cells growth, si-NC, pcDNA3.1, si-TNS1 or pcDNA3.1-TNS1 were transfected into A549 and H460 cells. The transfection efficiency was analyzed by RT-qPCR and Western blot (Figure 2A,B). Then, the effect of TNS1 on NSCLC cell growth was determined by MTT and flow cytometry. The results suggested that si-TNS1 remarkedly suppressed the proliferation and promoted the apoptosis of A549 and H460 cells, while pcDNA3.1-TNS1 performed the reverse functions (Figure 2C,D).

### TNS1 promotes NSCLC cell metastasis

**Figure 3:**
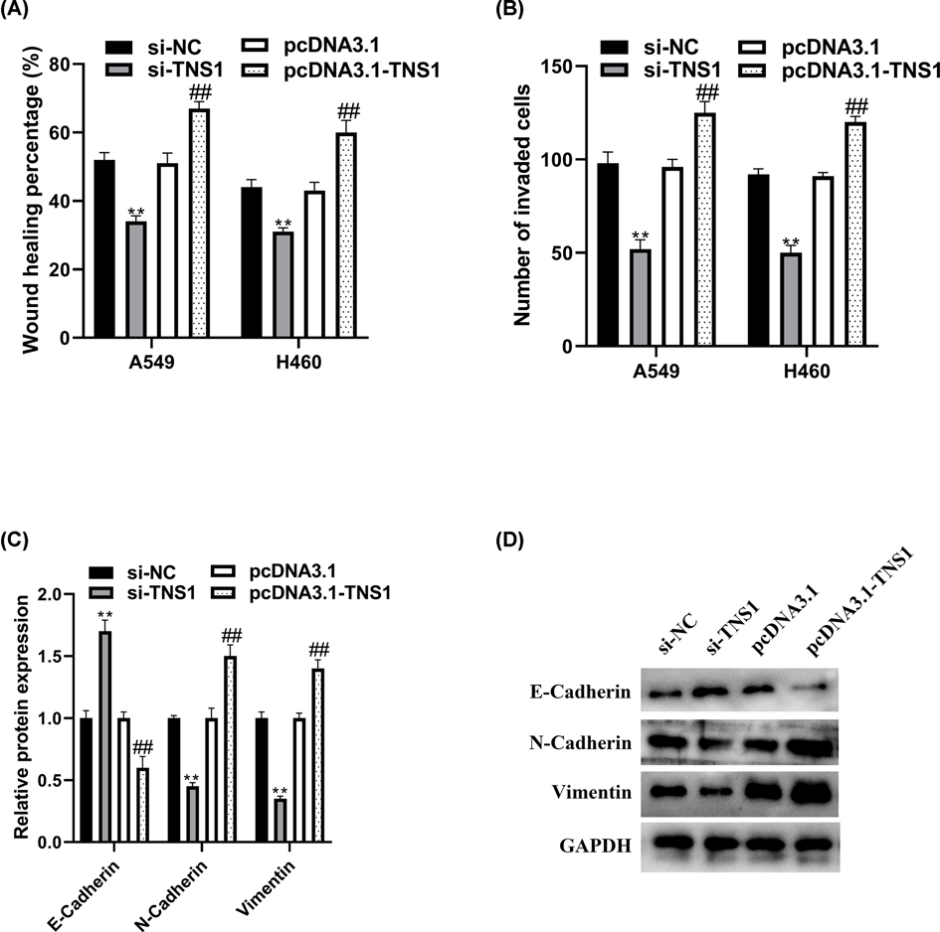
TNS1 suppresses cell metastasis of NSCLC cells (**A**) Wound-healing assay was employed to evaluate cell migration level. (**B**) Cell invasion ability was analyzed by transwell assay. (**C,D**) The protein expression of E-cadherin, N-cadherin and vimentin were assessed by Western blot. **P<0.01 compared with si-NC. ^##^*P*<0.01 compared with pcDNA3.1.

To further explore the function of TNS1 in NSCLC, wound-healing, transwell assays and Western blot were performed. Results showed that the migration and invasion levels of NSCLC cell lines were significantly decreased after si-TNS1 transfection while prompted by pcDNA3.1-TNS1 (Figure 3A,B). Western blot was recruited to analyze the effect of TNS1 on EMT marker proteins’ expression. The results showed that si-TNS1 up-regulated the expression of epithelial markers, namely, E-cadherin, and inhibited the expression of mesenchymal markers, including N-cadherin and vimentin. pcDNA3.1-TNS1 promoted EMT process (Figure 3C,D).

### pcDNA3.1-TNS1 promotes NSCLC progression by activating Akt/mTOR/RhoA signaling

**Figure 4:**
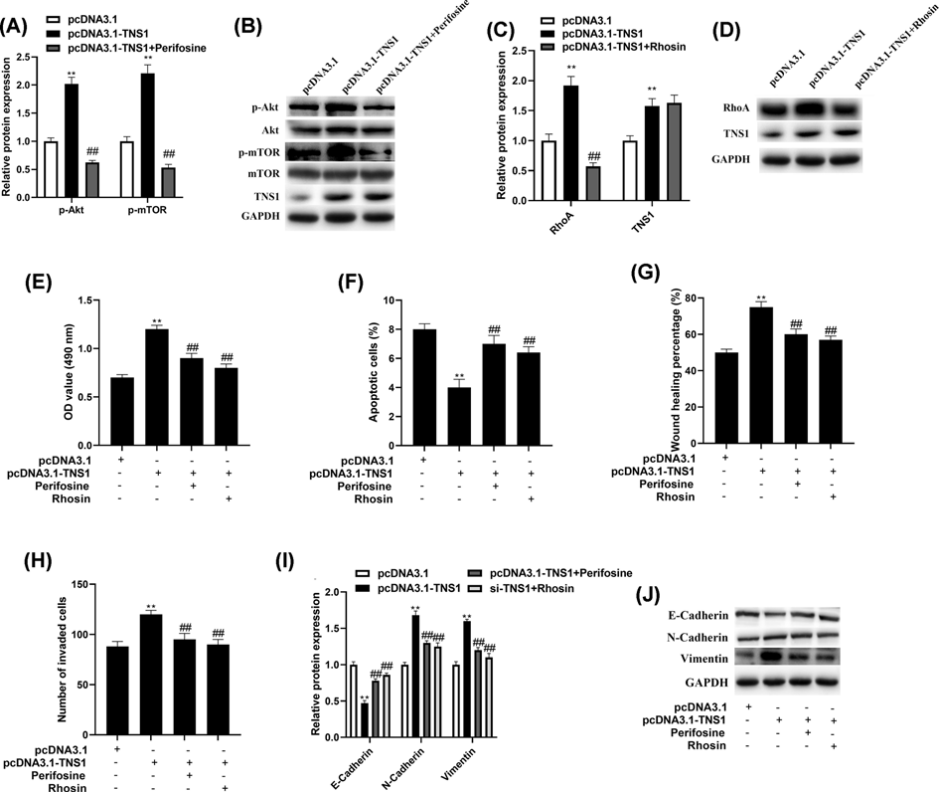
TNS1 promotes NSCLC progression by acting AKT/mTOR/RhoA signaling (**A,B**) p-Akt/Akt and p-mTOR/mTOR expression was determined by Western blot analysis. (**C,D**) Pull-down assay was employed to determine the activity of RhoA. (**E,F**) Cell growth was assessed through MTT assay and flow cytometry. (**G**–**J**) Wound-healing, transwell assay and Western blot were used to measure the metastasis of A549. **P<0.01 compared with pcDNA3.1, ^##^*P*<0.01 compared with pcDNA3.1-TNS1.

Next, we explored the probable mechanisms involved in the TNS1-mediated cell growth and metastases. Akt/mTOR and RhoA signaling pathway are both critical in lung cancer development [24,25], which are reported to be regulated by TNS1 [26,27]. The results implied that pcDNA3.1-TNS1 increased p-AKT/AKT ratio and p-mTOR/mTOR ratio. Perifosine (50 nM, Selleck Chemicals LLC), Akt inhibitor, could effectively lower the p-AKT/AKT ratio and p-mTOR/mTOR ratio while Rhosin (25 μM, Merck Millipore), RhoA inhibitor, suppressed RhoA (Figure 4A–D). Furthermore, the induction of cell growth induced by pcDNA3.1-TNS1 was inhibited by perifosine and Rhosin (Figure 4E,F). Consistently, perifosine and Rhosin significantly reversed pcDNA3.1-TNS1-promoted migration, invasion and EMT of A549 cells (Figure 4G–J). To conclude, these results elucidated that pcDNA3.1-TNS1 might advance the growth and metastasis of NSCLC cells by modulating AKT/mTOR/RhoA pathway.

### TNS1 is a direct target of miR-152 in NSCLC

**Figure 5:**
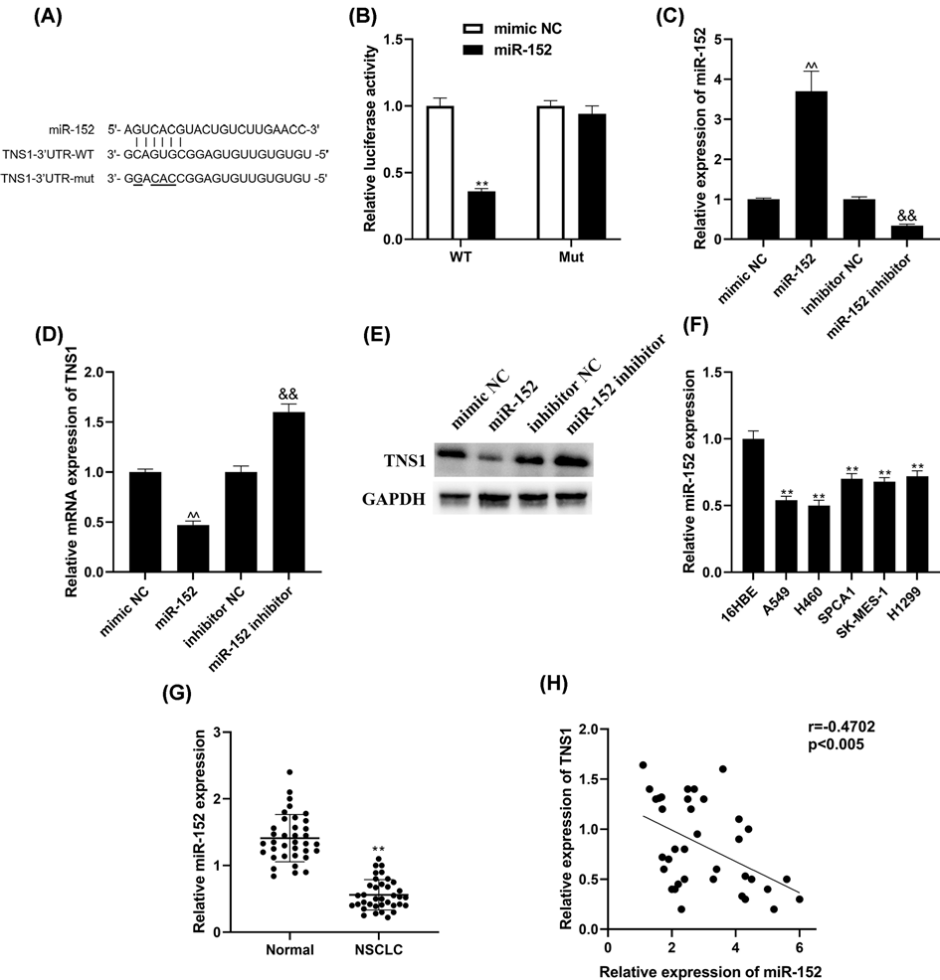
TNS1 is a target gene of miR-152 (**A**) The potential miR-152 binding sites of TNS1 3′-UTR and the mutant. (**B**) Dual-luciferase reporter assay was recruited to identify whether miR-152 targeted TNS1. **P<0.01 vs. mimic NC. (**C**) miR-152 expression was detected through RT-qPCR. ^ΛΛ^*P*<0.01 vs. mimic NC. ^&&^*P*<0.01 compared with inhibitor NC. TNS1 mRNA (**D,E**) protein expression was analyzed by RT-qPCR and Western blot. ^ΛΛ^*P*<0.01 vs. mimic NC. ^&&^*P*<0.01 compared with inhibitor NC. (**F,G**) miR-152 level in NSCLC cells and tissues was determined through Western blot. **P<0.01 vs. adjacent normal lung tissues or 16HBE cells. (**H**) Correlation between miR-152 and TNS1 expression in NSCLC tumor tissues was determined through Spearman’s rank correlation analysis.

Prediction results showed that TNS1 was a potential target of miR-152. Furthermore, the 3′-UTR of TNS1 has a putative seed region for miR-152 binding (Figure 5A). Luciferase reporter assay result indicated that miR-152 evidently suppressed the luciferase activity of the reporter containing wild-type TNS1 3′-UTR, while mutation of miR-152-binding sites in the TNS1 3′-UTR inhibited the regulatory effect of miR-152 on luciferase activity (Figure 5B). In addition, both mRNA and protein expression of TNS1 were significantly down-regulated by miR-152 mimics, while promoted by miR-152 inhibitor (Figure 5C–E). Above data suggested that miR-152 directly bound to TNS1 and regulated the expression of TNS1. RT-PCR analysis suggested that miR-152 was suppressed in both NSCLC cells and tissues (Figure 5F,G). Noticeably, correlation analysis demonstrated that in NSCLC tumor tissues, miR-152 expression was negatively correlated with TNS1 expression (Figure 5H).

### miR-152/TNS1 axis inhibits NSCLC progression

**Figure 6:**
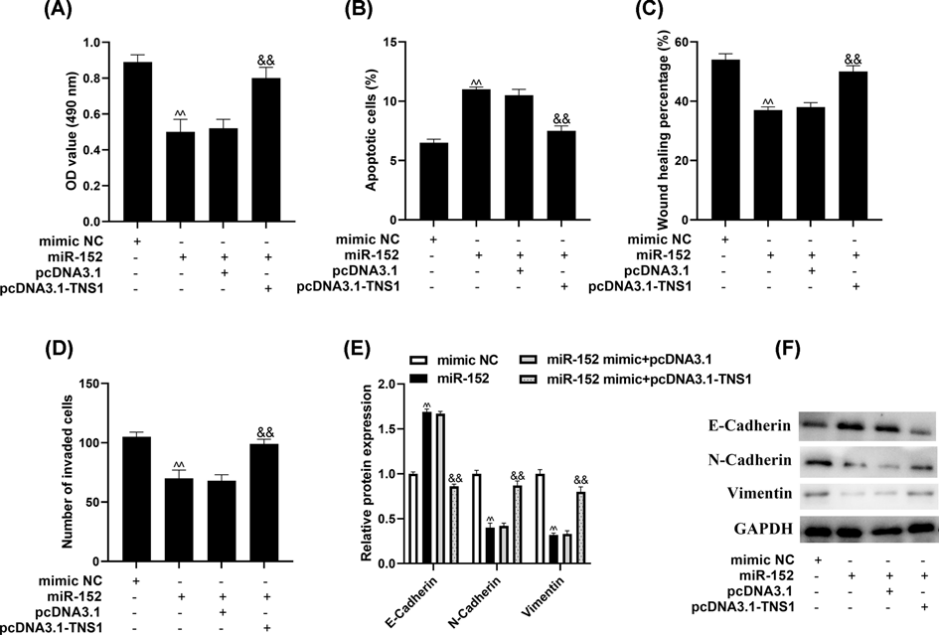
miR-152/TNS1 axis inhibits NSCLC progression The proliferation and apoptosis of A549 were analyzed via (**A**) MTT assay and (**B**) flow cytometry. The metastasis capacity of A549 was determined through (**C**) wound-healing, (**D**) transwell assays and Western blot. ^ΛΛ^*P*<0.01 compared with mimic NC, ^&&^*P*<0.01 compared with miR-152 + pcDNA3.1.

Then, the relationship of miR-152 and TNS1 in NSCLC development were evaluated. The results showed that TNS1 could reverse the effects of miR-152 on cell growth (Figure 6A,B) and metastasis of A549 cells (Figure 6C–F).

## Discussion

TNS1 has the ability of actin cross-linking, and binding to actin filaments through various signaling pathways. Furthermore, TNS1 participated in the disassembly of focal adhesions [5], integrin translocation [28], maintenance of normal renal function [29] and wound healing [30]. More importantly, many researches have indicated that TNS1 correlated with lung function and disease [31–33]. For example, variants in TNS1 were related to chronic obstructive pulmonary disease [34]. TNS1 was strongly up-regulated during myofibroblast differentiation and in fibrotic lung [35]. It has been reported that expression of TNS1 was increased in colorectal cancer [8], breast cancer [36] and acute myeloid leukemia [10]. In lung cancer, it was found that TNS1 expression was significantly higher in NSCLC tissues and cells than normal. Furthermore, Kaplan–Meier survival analysis results indicated that higher expression of TNS1 was highly correlated with poor patient prognosis in lung cancer, which was in accordance with the research in colorectal cancer of Zhou et al. [8]. All these suggested that TNS1 might be involved in the progression of NSCLC. TNS1 is reported to play different roles in different cancers. For example, TNS1, as an oncogene, participated in proliferation and invasion in colorectal cancer [7,8], while acting as a tumor suppressor in breast cancer cells [37]. Our results indicated that TNS1 could promote cell proliferation and inhibit apoptosis. Meanwhile, we also observed the facilitation effect of pcDNA3.1-TNS1 on NSCLC cell migration, EMT and invasion. All these were consistent with the research in colorectal cancer [38,39].

AKT/mTOR pathway plays crucial roles in multiple biological processes, including cell proliferation, migration, cell cycle progression and apoptosis in breast cancers, NSCLC, colorectal cancers and other human cancers [40–44]. AKT is the significant molecule downstream of PI3K which participates in cell growth and survival. Once AKT was phosphorylated, numerous downstream targets in cytoplasm and nucleus would activate advance cell growth and survival. Activated mTOR, the key substrate of AKT, could active eukaryotic initiation factor 4E and lead to the proliferation of cells. Furthermore, multiple researches have reported that the Rho/Rho-kinase signaling pathway noticeably participated in cancer invasion, growth and metastasis, and Rho guanosine triphosphate hydrolases are associated with Ras-mediated oncogenic transformation [45,46]. Inhibiting the Rho/Rho-kinase pathway curbs NSCLC cells migration and invasion [47,48]. In this research, pcDNA3.1-TNS1 could remarkedly increase the phosphorylation of AKT and mTOR and RhoA activity in NSCLC cells, and significantly promote cell growth and metastasis through AKT/mTOR/RhoA pathway.

Through luciferase reporter assay and correlation analysis, it was confirmed that miR-152 targeted TNS1 and reversely correlated. miR-152 belongs to the miR-148/152 family which is composed of miR-148a/b and miR-152. miR-152 is a tumor suppressive miRNA and inhibited in various cancers. Aberrant expression of miR-152 is believed to cause the malignant phenotype of various tumors [49]. It was reported that overexpressing miR-152 could suppress proliferation and promote apoptosis through targeting of DNMT1 in ovarian cancer cells [50]. It was demonstrated that miR-152 overexpression also curbed the migratory and invasive abilities of prostate cancer cells [51]. miR-152 regulates growth and metastases of NSCLC cells through targeting ADAM17 and FGF2 [49,52]. Our researches showed that miR-152 also affects cell growth and metastasis by regulating TNS1/Akt/mTOR/RhoA in NSCLC.

In this research, we found that TNS1 was significantly up-regulated in NSCLC patients and related to the growth and metastasis of NSCLC. Employing NSCLC cell lines, we further determined that TNS1 boosted the proliferation, apoptosis, migration, invasion and EMT of NSCLC cells. Besides, miR-152 targeted TNS1 and TNS1 facilitating NSCLC progression by curbing AKT/mTOR/RhoA signaling. This is the first study reporting TNS1 regulated NSCLC cells progression which might be conducive to develop treatment therapy for NSCLC.

## Abbreviations

ADAM17: A Disintegrin And Metalloprotease 17
EMT: epithelial–mesenchymal transition
FITC: fluorescein isothiocyanate
FGF2: fibroblast growth factor 12
GAPDH: glyceraldehyde 3-phosphate dehydrogenase
miRNA: microRNA
MTT: 3-(4,5)-dimethylthiahiazo (-z-y1)-3,5-di- phenytetrazoliumromide
NSCLC: non-small cell lung cancer
Rho: Ras homolog
RIPA: Radio-Immunoprecipitation Assay
TNS1: tensin 1
3′-UTR: 3′-untranslated region
